# Elucidation of physico-chemical principles of high-density lipoprotein–small RNA binding interactions

**DOI:** 10.1016/j.jbc.2022.101952

**Published:** 2022-04-18

**Authors:** Danielle L. Michell, Ryan M. Allen, Ashley B. Cavnar, Danielle M. Contreras, Minzhi Yu, Elizabeth M. Semler, Clark Massick, Chase A. Raby, Mark Castleberry, Marisol A. Ramirez, Wanying Zhu, Linda May-Zhang, Anca Ifrim, John Jeffrey Carr, James G. Terry, Anna Schwendeman, Sean S. Davies, Quanhu Sheng, MacRae F. Linton, Kasey C. Vickers

**Affiliations:** 1 Department of Medicine, Vanderbilt University Medical Center, Nashville, Tennessee, USA; 2 Department of Pharmaceutical Sciences, University of Michigan, Ann Arbor, Michigan, USA; 3 Department of Molecular Physiology and Biophysics, Vanderbilt University, Nashville, Tennessee, USA; 4 Department of Biostatistics, Vanderbilt University Medical Center, Nashville, Tennessee, USA; 5 Department of Pharmacology, Vanderbilt University, Nashville, Tennessee, USA; 6 Department of Radiology and Radiological Sciences, Vanderbilt University Medical Center, Tennessee, USA

**Keywords:** HDL, tRNA, atherosclerosis, apolipoprotein A-I, RNA binding protein, AS, antisense, apoA-I, apolipoprotein A-I, BMDM, bone marrow–derived macrophages, CAC, coronary artery calcium, dsRNA, double-stranded RNA, DGUC, density-gradient ultracentrifugation, EMSA, electrophoretic mobility shift assay, HDL, high-density lipoprotein, IsoLG, isolevuglandin, LNA, locked-nucleic acid, miRNA, microRNA, MST, microscale thermophoresis, ONE, 4-oxo-nonenal, POPC, 1-palmitoyl-2-oleoyl-sn-glycero-3-phosphocholine, PC, phosphatidylcholine, PBMC, peripheral blood mononuclear cell, rDRs, rRNA-derived sRNAs, rHDL, reconstituted HDL, sHDL, synthetic HDL, sRNAs, small RNAs, TC, total cholesterol, tDRs, tRNA-derived sRNAs, tRH, tRNA-derived halves, tRF, tRNA-derived fragment

## Abstract

Extracellular small RNAs (sRNAs) are abundant in many biofluids, but little is known about their mechanisms of transport and stability in RNase-rich environments. We previously reported that high-density lipoproteins (HDLs) in mice were enriched with multiple classes of sRNAs derived from the endogenous transcriptome, but also from exogenous organisms. Here, we show that human HDL transports tRNA-derived sRNAs (tDRs) from host and nonhost species, the profiles of which were found to be altered in human atherosclerosis. We hypothesized that HDL binds to tDRs through apolipoprotein A-I (apoA-I) and that these interactions are conferred by RNA-specific features. We tested this using microscale thermophoresis and electrophoretic mobility shift assays and found that HDL binds to tDRs and other single-stranded sRNAs with strong affinity but did not bind to double-stranded RNA or DNA. Furthermore, we show that natural and synthetic RNA modifications influenced tDR binding to HDL. We demonstrate that reconstituted HDL bound to tDRs only in the presence of apoA-I, and purified apoA-I alone were able to bind sRNA. Conversely, phosphatidylcholine vesicles did not bind tDRs. In summary, we conclude that HDL binds to single-stranded sRNAs likely through nonionic interactions with apoA-I. These results highlight binding properties that likely enable extracellular RNA communication and provide a foundation for future studies to manipulate HDL–sRNA interactions for therapeutic approaches to prevent or treat disease.

High-density lipoproteins (HDLs) have many beneficial properties which antagonize inflammation, infection, metabolic dysfunction, and disease (1, 2). HDL is the smallest class of lipoproteins (7–12 nm in diameter), and the HDL pool is composed of a compendium of subspecies with different sizes, shapes, and functions (1). Apolipoprotein A-I (apoA-I), the primary structural and functional protein of HDL, is secreted from hepatocytes and intestinal enterocytes (3). Upon secretion, apoA-I acquires phospholipids and free cholesterol from cells to form nascent pre-β discoidal HDL particles (4, 5). Further acceptance and esterification of free cholesterol cargo results in the formation of mature spherical particles containing a hydrophobic core with a phospholipid monolayer shell. HDL particles are dynamic and continuously load and unload cargo with interacting cells and other lipoproteins. The HDL pool contains >215 different proteins; however, apoA-I accounts for approximately 70% of HDL total protein mass https://homepages.uc.edu/∼davidswm/HDLproteome.html (6). In addition to proteins and diverse bioactive lipids, HDL also transport many classes of small noncoding RNAs (7, 8) in both plasma and biofluids (*e.g.*, lymph) and have been shown to transport and deliver functionally active microRNAs (miRNAs) to recipient cells where they participate in gene regulation networks (9, 10, 11, 12). We reported that HDL’s anti-inflammatory capacity in endothelial cells was found to be mediated, in part, by HDL delivery of miR-223-3p to recipient human coronary artery endothelial cells and silencing of intercellular adhesion molecule 1 (9). The HDL–miRNA profile is significantly altered in multiple diseases, including familial hypercholesterolemia, atherosclerosis, uremia, diabetes, and obesity (8, 13, 14, 15, 16). HDL–miRNAs have also been reported to be altered by diet and specific dietary components (17, 18, 19). To date, most investigation of extracellular small RNAs (sRNAs) on lipoproteins has been limited to miRNAs; however, miRNAs only represent a small fraction of sRNAs on HDL and other classes of sRNA warrant investigation (7). Recently, we profiled HDL–sRNAs in mice using high-throughput sRNA sequencing, and many classes of host and nonhost sRNAs were identified on circulating HDL (7). Results showed that HDL also transport tRNA-derived sRNAs (tDRs) and rRNA-derived sRNAs (rDRs) (7). Furthermore, we found that mouse HDLs are highly enriched with nonhost microbial sRNAs which likely originate from bacteria and fungi in the microbiome, environment, and/or diet.

HDL–sRNAs are likely single-stranded and generally <60 nts in length (7, 8). HDLs have been found to accept sRNAs from multiple cell types, including miR-223-3p from macrophages and neutrophils, and miR-375-3p from pancreatic beta cells (8, 20, 21). Nevertheless, the mechanism by which HDL acquires sRNAs from donor cells and the underlying biochemistry of HDL–sRNA binding have remained elusive. Studies suggest that oligonucleotides can associate with zwitterionic lipids, *e.g.*, phosphatidylcholine (PC) (22, 23, 24, 25), but it is currently unknown if natural PC within HDL’s shell mediate its transport of sRNA. Results from HDL delipidation studies suggest that miRNAs may bind to HDL proteins as opposed to phospholipids (13, 26); however, to date, a candidate protein on HDL with RNA binding capacity has not been identified. Here, we tested the hypothesis that apoA-I is a novel RNA binding protein for single-stranded sRNAs, specifically tDRs. Microscale thermophoresis (MST) and electrophoretic mobility shift assay (EMSA) data suggest that apoA-I is a novel ribonucleoprotein that likely confers the ability of HDL to bind RNA, rather than the lipid cargo of the particle. MST approaches were used to define the impact of base modifications and the 2′ hydroxyl group on HDL–sRNA binding. These results support a mechanism by which HDL binds sRNAs, including tDRs, and reveal the physicochemical properties that influence HDL–sRNA binding.

## Results

### Human HDL transports diverse sRNA classes (Fig. 1)

**Figure 1:**
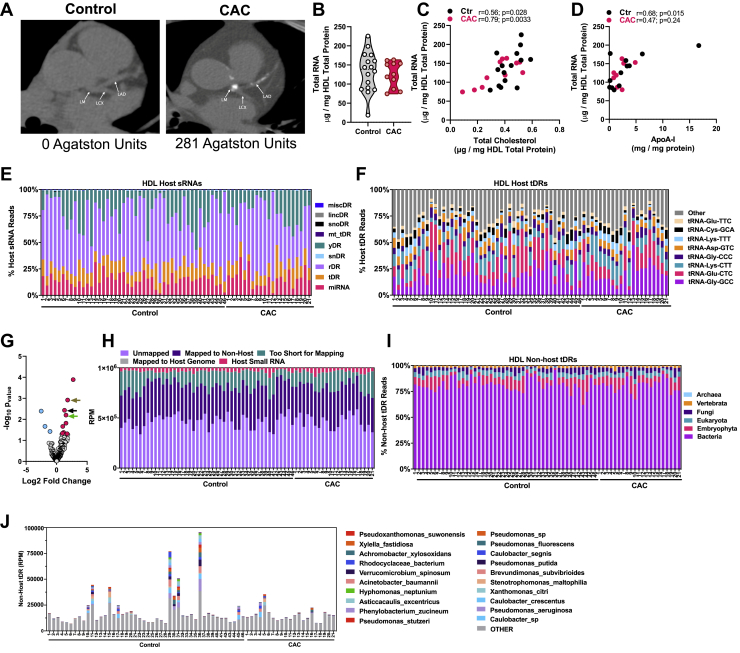
**Distribution of small noncoding RNAs on human HDL.***A*, noncontrast CT (computed tomography) scans from representative control and individual with calcified coronary atheroma and CAC score >0. *Left*, CAC=0; *right*, CAC=281. *Arrows* indicate LAD (left anterior descending coronary artery), LCX (left circumflex artery), and LM (left main coronary artery). *B*, total RNA content as determined by direct SYTO RNASelect staining (50 μM) on DGUC-HDL from control (n = 16) and CAC subjects (n = 11), normalized to total protein. Violin plot showing median and quartile ranges. *C*, correlation of total RNA content from (*B*) and total cholesterol levels normalized to total protein. *D*, correlation of Total RNA content (*B*) and apoA-I protein (ELISA) normalized to total protein. Results from sRNA-seq analysis of DGUC-HDL in control (n = 46) and CAC (n = 21) subjects. *E*, distribution of host sRNAs abundance as percentage of total host reads. *F*, percentage of host tDR by anticodon. *G*, differentially altered tDRs reads. Altered tDR-GlyGCC reads indicated with *arrows*, *brown*=tDR-GlyGCC-30; *black*=tDR-GlyGCC-33; *green*=tDR-GlyGCC-32. *H*, reads per million (RPM) identified as host, nonhost, genome, unmapped, or too short for conservative mapping with TIGER (7). *I*, alignment to nonhost (nonhuman) tDR database grouped by taxa. *J*, alignment to top nonhost tDR bacteria species. CAC, coronary artery calcium; DGUC, density-gradient ultracentrifugation; HDL, high-density lipoprotein; tDR, tRNA-derived sRNA; sRNAs, small RNAs.

The determination of overall total RNA content on human HDL particles has previously been challenging due to technical limitations that require RNA isolation and downstream quantitation of individual candidate sRNAs of prior knowledge. To overcome these barriers, total RNA levels were quantified in intact human HDL with SYTO RNASelect, a fluorescence-based membrane-penetrating RNA dye that does not require RNA isolation (27). HDLs were isolated from healthy controls (control, mean=0, N = 46) and subjects with indications of atherosclerosis (coronary artery calcium [CAC], mean=61, n = 21) (Fig. 1*A* and Table S1). Based on an oligoribonucleotide standard curve, HDL particles were determined to transport about 128 μg of total RNA per mg HDL total protein for healthy control subjects (n = 16) and 125 μg total RNA per mg HDL total protein for CAC subjects (n = 11) (Fig. 1*B*). In both control and CAC subjects, density-gradient ultracentrifugation (DGUC)-HDL total cholesterol (TC) levels were significantly correlated to HDL–RNA (Fig. 1*C*). HDL–apoA-I protein levels were also found to be significantly correlated to HDL–RNA levels in control subjects, but not CAC subjects (Figs. 1*D*, S1, *A* and *B*).

Host miRNAs are only a minor fraction of HDL–sRNA cargo and further investigation of non-miRNA classes for content and function are warranted. tDRs are associated with cellular stress and likely function as sRNA regulators of cell survival and protein synthesis (28, 29, 30, 31, 32, 33, 34, 35, 36, 37); however, the functional impact of extracellular HDL–tDRs remains to be determined. To define the human HDL–tDR profile and quantify differences in HDL–tDR levels in atherosclerosis (CAC *versus* control), sRNA-seq and informatics (TIGER pipeline) were performed (7). Preprocessed reads from HDL–sRNA-seq were aligned to the human reference genome (hg19) as well as to additional parent tRNA transcripts not present in the reference genome (7). Special considerations were made for tRNA nomenclature, multimapping, and terminal 3′ CCA masking; and tDR read counts were tabulated for parent tRNAs at the amino acid anticodon level, as well as individually at the read level (7). The most abundant class of host (human) sRNAs on DGUC-HDL were rDRs, followed by yDRs (Y RNA–derived sRNAs), miRNAs, and tDRs (Fig. 1*E*). Differential expression analyses (DEseq2) were used to identify altered HDL–sRNAs and multiple miRNAs, rDRs, and tDRs were found to be significantly increased or decreased on HDL from human atherosclerotic subjects (CAC) compared to control subjects (Fig. S1*C* and Tables S2–S5). Summary counts for parent tRNA-GlyGCC were the highest on human HDL, followed by tRNA-GluCTC and tRNA-LysCTT (Fig. 1*F* and Table S2). Multiple tDRs were observed to be significantly altered in human atherosclerosis at the individual sRNA level, as opposed to the parent transcript level (Table S3). For example, HDL levels of tDR-GlyGCC-30, a 30 nt tDR processed from the 5′ half of parent tRNA-GlyGCC, were increased 3.5-fold (*p* = 0.0012) on HDL from CAC subjects compared to control subjects (Fig. 1*G* and Table S3).

In our previous study, mouse HDL were demonstrated to transport many nonhost sRNAs, including microbial sRNAs, not from laboratory contamination, but indeed from bacteria and fungi in the microbiome and environment (7). To determine if human HDL also transport microbial sRNA, all nonhost reads were further aligned to curated nonhost genomes, including bacteria, fungi, and viruses. Strikingly, we found that DGUC-HDL from human plasma are also highly enriched with nonhost microbial sRNAs (Fig. 1*H*). To determine if HDL transports nonhuman tDRs, nonhost reads were further aligned to a database of tRNA transcripts (GtRNAdb), and human HDL were observed to carry many bacterial tDRs (Fig.1*I* and Table S6). Interestingly, many nonhost tDRs aligned to bacterial tRNAs curated in the GtRNAdb database (38), as reported at the species (count) level (Fig. 1*J* and Table S6). Nonetheless, host tDR-GlyGCC-30 was still found to be one of the most altered sRNAs on HDL in subjects with CAC. These results support the notion that human HDL transports high levels of sRNA in circulation, and HDL–sRNA levels are correlated to HDL-cholesterol and apoA-I levels. Moreover, results support the notion that human HDL transports specific tDRs which are altered in atherosclerosis; however, the mechanism by which HDL binds to tDRs is unknown.

### Immune cell export of tDRs to HDL (Fig. 2)

**Figure 2:**
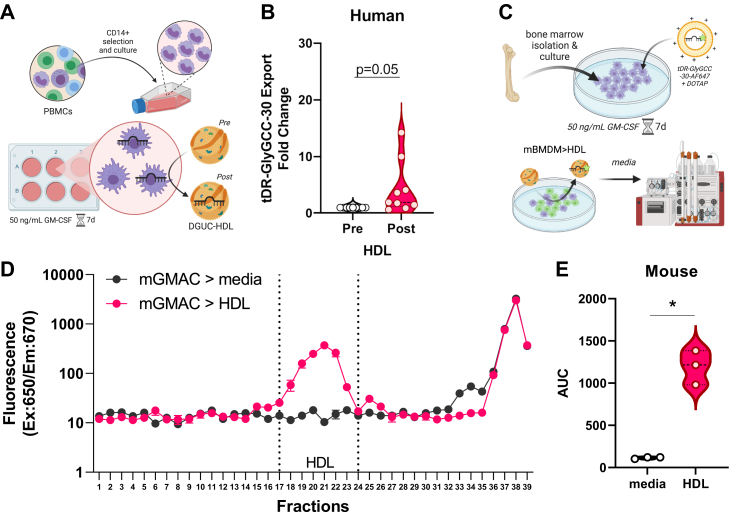
**Macrophage export of sRNAs to HDL.***A*, schematic representation of experimental design used in (*B*). *B*, HDL tDR-GlyGCC-30 expression by real-time PCR before (Pre) and after (Post) exposure to human GM-CSF differentiated macrophages. Violin plot showing median and quartile ranges, n = 9. Mann–Whitney nonparametric test. ∗*p* < 0.05. *C*, schematic representation of experimental design used in (*D*–*E*). *D*, fluorescence signal of exported tDR-GlyGCC-30 (AF647) from mouse bone marrow–derived macrophages (mGMAC) to serum free media (mGMAC>media) or DGUC-HDL (mGMAC>HDL) across SEC fractions (1.5 ml), data are mean ± SD, n = 3. *E*, area under the curve (AUC) of HDL region from (*D*). Violin plot showing median and quartile ranges. Unpaired *t* test. ∗*p* < 0.05. Schematics created with BioRender.com. DGUC, density-gradient ultracentrifugation; HDL, high-density lipoprotein; tDR, tRNA-derived sRNA; sRNAs, small RNAs.

We have previously observed that macrophages and other immune cells readily export miRNAs to HDL *in vitro* (8, 20); however, it is unknown if macrophages also export other sRNA classes to HDL. Cellular tDR changes are associated with stress and macrophages within the atherosclerotic lesion are under both environmental (local tissue inflammation) and metabolic stresses (cholesterol-loading endoplasmic reticulum stress) (29, 39). To determine if macrophages export tDRs to HDL, CD14+ human peripheral blood mononuclear cells (PBMCs) were isolated from whole blood, differentiated to macrophages for 7 days with GM-CSF (50 ng/ml), and incubated with 1 mg/ml DGUC-HDL for 24 h in serum-free media (Fig. 2*A*). HDL were repurified from culture media by size-exclusion chromatography (SEC), and total RNA was isolated from equal protein levels (0.2 mg HDL total protein) of HDL preincubation and postincubation with macrophages. To quantify tDR-GlyGCC-30 export, qPCR with custom probes were performed, and we observed an increase in HDL-tDR-GlyGCC-30 levels on post-HDL compared to pre-HDL (Fig. 2*B*). To confirm macrophage HDL–tDR export, mouse bone marrow cells were differentiated to bone marrow–derived macrophages (BMDM) with GM-CSF for 7 days, transiently-transfected for 24 h with fluorescence-labeled tDR-GlyGCC-30-AF647, washed, and incubated with accepting HDL (1 mg/ml) for 24 h (Fig. 2*C*). BMDM culture media was collected and separated by SEC, and HDL-tDR-GlyGCC-30 export was determined by the levels of fluorescence (tDR-GlyGCC) in HDL (SEC) fractions. We observed a significant increase in fluorescence signal in fractions corresponding to HDL compared to media from wells that did not receive accepting HDL (Fig. 2, *D* and *E*). These results suggest that primary macrophages in both humans and mice have the capacity to export tDRs, namely tDR-GlyGCC-30, to HDL *in vitro*. Although the mechanism by which HDL accepts tDRs from macrophages is unknown, HDL may have the capacity to bind to free, unprotected extracellular tDRs exported from macrophages.

### Differential sRNA binding affinities to HDL (Fig. 3)

**Figure 3:**
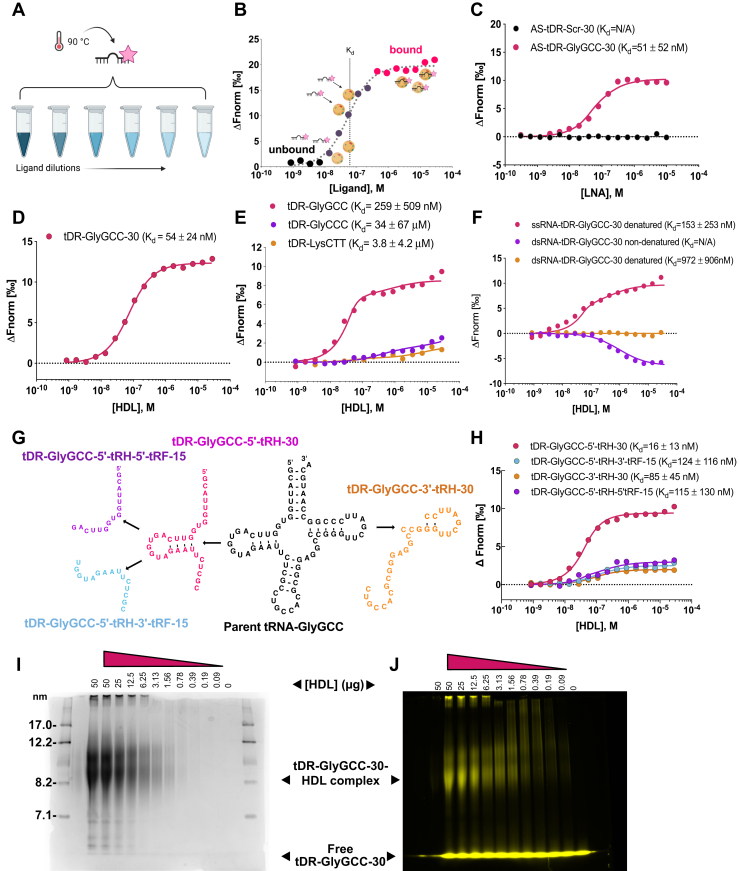
**tDR-GlyGCC binding affinity to HDL by microscale thermophoresis.***A*, setup of microscale thermophoresis (MST). Fluorescently labeled (AF647) target (*e.g.*, single stranded sRNA oligos) were heat denatured (90 °C for 2 min) and combined (1:1) with serial ligand dilutions (*e.g.*, DGUC-HDL). Oligo-ligand mix were then loaded in glass capillaries, and MST was performed where an infrared laser heats the complex to create a small temperature gradient. sRNAs with HDL will demonstrate slower movement compared to free sRNA molecules. The movement of the fluorescent sRNA is then tracked. *B*, example of a binding curve depicting difference between unbound sRNAs and bound sRNA molecules thermophoretic movement, where Fnorm is the normalized fluorescence against the ligand concentration, ΔFnorm is the baseline corrected normalized fluorescence. Data were fitted to the MST (K_d_) model. Mean MST analysis from triplicate assays are presented, K_d_ are mean ± SD. *C*, MST analysis of interactions between tDR-GlyGCC-30 and a scrambled antisense (AS) oligonucleotide (AS-Scr) or a complementary antisense to tDR-GlyGCC-30 (AS-tDR-GlyGCC-30). *D*, MST binding curves of tDR-GlyGCC-30 and DGUC-HDL. *E*, MST analysis of interactions between DGUC-HDL and tDR-GlyGCC-30, tDR-GlyCCC-30, or tDR-LysCTT-30. *F*, binding curves of DGUC-HDL to single-stranded (ssRNA) or double-stranded (dsRNA) tDR-GlyGCC-30 without (nondenatured) or with heat (90 °C 2 min) denaturing (denatured). *G*, schematic of tDR-GlyGCC fragments synthesized used in (*H*). *H*, MST analysis of interactions between DGUC HDL and tDR-GlyGCC fragments in (*G*). Electrophoretic mobility shift assay (EMSA) with fluorescently (AF546) labeled sRNA and DGUC-HDL. tDR-GlyGCC (AF546) was incubated with titrations of HDL (1:1) and crosslinked (@254 nm) for 15 min. Samples were loaded onto a 4–16% NativePAGE gel with 0.5× TBE running buffer. *I*, Coomassie of nondenaturing gel. Stokes radius (R_s_) of proteins standards shown. *J*, fluorescent (AF546) migration of tDR-GlyGCC-30 on gel with free oligo and oligo complexed to HDL titrations is shown. Schematics created with BioRender.com. DGUC, density-gradient ultracentrifugation; HDL, high-density lipoprotein; tDR, tRNA-derived sRNA; sRNAs, small RNAs.

To quantify the binding affinity of sRNAs to HDL, MST assays were completed with oligonucleotides labeled with a 3′ terminal fluorophore (Alexa Fluor 647) (Fig. 3, *A* and *B* and Table S7). As a positive control, the binding affinity of tDR-GlyGCC-30 to its complementary antisense (AS) sequence was tested, and strong affinity toward AS-GlyGCC-30 was observed (Fig. 3*C*). tDR-GlyGCC-30 binding to a control scrambled sequence (AS-Scr) was also assayed as a negative control, and tDR-GlyGCC-30 binding was not observed (Fig. 3*C*). To calculate HDL’s binding affinity to tDR-GlyGCC-30, multiple DGUC-HDL samples were tested with MST, and we found that HDL does indeed have strong affinity toward tDR-GlyGCC-30 (Fig. 3*D*). To determine if HDL has similar affinity to other tDRs of same length (30 nts), MST assays were used to calculate HDL affinities toward tDR-LysCTT-30 and tDR-GlyCCC-30, both of which were found to have reduced affinity toward HDL compared to tDR-GlyGCC-30 (Fig. 3*E*). These results suggest that HDL have preferential binding toward tDR-GlyGCC-30 compared to other HDL-tDRs. Based on informatics, HDL-sRNAs are likely single-stranded RNA (ssRNA); however, tDR-GlyGCC has been reported to form secondary hairpin structures that have been proposed to increase its stability and resistance to RNases in plasma (40, 41) but could also influence HDL association. To determine if HDL preferentially bind to ssRNA or double-stranded RNA (dsRNA), dsRNA-tDR-GlyGCC-30 was synthesized in ssRNA and dsRNA forms (Table S7) and tested with MST. Human DGUC-HDL again showed strong affinity to ssRNA-tDR-GlyGCC-30; however, HDL failed to bind to dsRNA-tDR-GlyGCC-30, suggesting that HDL may indeed prefer ssRNA over dsRNA, at least for tDR-GlyGCC-30 (Fig. 3*F*). Denaturing dsRNA-tDR-GlyGCC-30 prior to MST with heat to break apart the duplex was found to restore HDL binding (Fig. 3*F*). Parent tRNAs can also be cleaved to produce multiple forms of sRNAs originating from the 5′ or 3′ terminal ends, often referred to as tRNA-derived halves (tRHs) or tRNA-derived fragments (tRFs) (29, 30, 42, 43). Our candidate, tDR-GlyGCC-30, is a 5′-tRH likely cleaved by angiogenin in response to stress (29, 34, 36); however, other forms and lengths of tDR-GlyGCC can also be cleaved. To determine if HDL have strong affinity to other potential tDRs arising from the parent tRNA-GlyGCC, specific tDR-GlyGCC fragments were synthesized and tested for binding affinity using MST (Table S7). HDL bound strongly to all candidate tDR-GlyGCC of variable lengths and sequences; however, the strongest binding affinity for HDL was observed to be our candidate tDR-GlyGCC-5′tRH-30 compared to tDR-GlyGCC-3′-tRF-15, tDR-GlyGCC-3′tRH, and tDR-GlyGCC-5′tRF-15 (Fig. 3, *G* and *H*). These results agree with observations from sRNA-seq of human HDL, as positional alignment analyses of tDRs mapped to their parent tRNAs showed enrichment for 5′ tRHs over 3′ tRHs for GlyGCC (Fig. S2*A*).

In addition to MST, EMSA were used as an orthogonal method to demonstrate HDL’s binding affinity to tDR-GlyGCC-30. Briefly, tDR-GlyGCC-30-AF546 was cross-linked (UV, 254 nm) to serial dilutions of DGUC-HDL for 15 min. Bound and unbound tDR-GlyGCC-30/HDL complexes were separated by native polyacrylamide gel electrophoresis, and RNA binding was visualized with a fluorescence scanner (Fig. 3,*I* and *J*). No fluorescence/RNA signal was observed in the HDL only lane; however, tDR-GlyGCC-30-AF546 crosslinked to HDL resulted in a shift (increase) in an HDL size (bands between: 8.2 and 12.2 nm, Fig. 3,*I* and *J*) in a dose-dependent manner. Moreover, the band position of HDL–tDRGlyGCC-30 complex was not observed in the RNA only lane. Based on HDL titrations, the binding affinity of HDL to tDR-GlyGCC-30-AF546 was calculated (Fig. S2*B*), as previously described (44), and results from EMSA confirmed that HDL harbors strong affinity toward tDR-GlyGCC-30. These results support that HDL preferentially bind to ssRNAs and possess strong affinity to tDRs based on length and sequence.

### Biochemical properties of sRNAs that impact HDL binding (Fig. 4)

**Figure 4:**
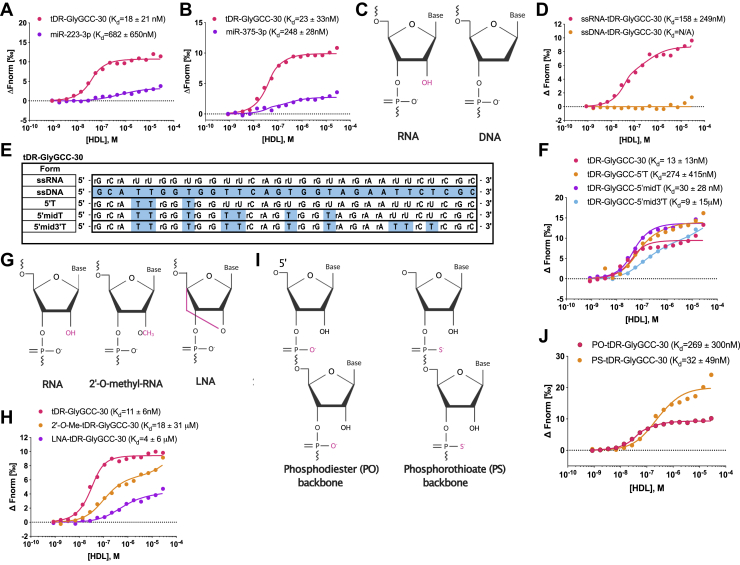
**Biochemical properties of tDR-GlyGCC binding to HDL using MST.** MST analysis of interactions between DGUC-HDL and (*A*) miR-223-3p or (*B*) miR-375-3p. *C*, structure of ribonucleotides (RNA) and deoxyribonucleotides (DNA). Hydroxyl group on RNA highlighted. *D*, representative MST binding curves of single-stranded RNA (ssRNA) or single-stranded DNA (ssDNA) of tDR-GlyGCC-30. *E*, synthesized tDR-GlyGCC-30 sequences of ssRNA, ssDNA, and rU>T mutations: at the 5′ terminal end (5′T), the 5′ terminal end and middle (5′midT), and the entire oligonucleotide (5′mid3′T). *F*, MST binding curves of DGUC-HDL with tDR-GlyGCC-30 and its rU>T mutations from (*E*). *G*, structure of RNA with 2′-O-methylation (2′-O-Me) or locked-nucleic acid (LNA) which harbors a bridge between 2′ and 5′ positions of the base ring. Modifications highlighted. *H*, MST binding curves of DGUC-HDL and tDR-GlyGCC-30 with either 2′-O-Me or LNA modifications. *I*, structure of RNA with phosphodiester (PO) or phosphorothioate (PS) backbones. *J*, MST binding curves of DGUC-HDL and tDR-GlyGCC-30 with either PO or PS backbones. Binding affinities (K_d_) of mean MST analysis from triplicate assays are presented, K_d_ are mean ± SD. Structures created with BioRender.com. DGUC, density-gradient ultracentrifugation; HDL, high-density lipoprotein; MST, microscale thermophoresis; tDR, tRNA-derived sRNA.

To calculate the binding affinity of DGUC-HDL to other classes of sRNAs present on HDL, candidate miRNAs were tested with MST. We have previously reported that miR-223-3p and miR-375-3p are abundant on circulating HDL (8, 20, 21). MST assays here confirmed that HDL binds to both miR-223-3p and miR-375-3p with strong affinities (Fig. 4, *A* and *B*). To further understand the biochemical and physical properties that influence HDL’s association to oligonucleotides, we examined the 2′ position of the ribose in ribonucleotides as this position distinguishes RNA from DNA. To determine the affinity of HDL to DNA, which lacks a hydroxyl group at the 2′ position, ssDNA-tDR-GlyGCC-30 was synthesized (Table S2 and Fig. 4*E*) and tested for HDL binding with MST. Results showed that HDL readily bound to the ssRNA form but failed to bind to the DNA form of tDR-GlyGCC-30 (Fig. 4, *C*–*E*). ssDNA-tDR-GlyGCC-30 harbored DNA bases along the length of the tDR-GlyGCC oligonucleotide, including thymines in lieu of uracils at 10 positions (Table S7 and Fig. 4*E*). These results suggest that HDL binding to sRNA is enhanced by either the 2′ hydroxy group present on RNA or by uracil present in sRNA sequences. To determine if uracil facilitates sRNA binding to HDL, ssRNA form of tDR-GlyGCC-30 was synthesized with rU>T mutations at regional positions along the oligonucleotide. Mutations were created at the 5′ terminal end (5′T), 5′ terminal end + middle (5′midT), or the entire oligonucleotide (5′mid3′T) (Table S7 and Fig. 4*E*). Based on MST assays, each of these three designs were observed to bind to HDL; however, the 5′T harboring the least amount of thymines showed the strongest binding affinity toward HDL. HDL’s affinity toward the 5′T mutant form of tDR-GlyGCC-30 was less than the native form of tDR-GlyGCC-30 (Fig. 4*F*). These results support that sRNA uracil content is associated with increased binding affinity to HDL.

To investigate the impact of the 2′ position on HDL–sRNA binding, tDR-GlyGCC-30 was synthesized with an O-linked methyl group at the 2′ position in place of the normal hydroxyl group, and MST assays found that 2-O-methylation reduced HDL binding affinity (Fig. 4, *G* and *H* and Table S7). Locked-nucleic acids (LNAs) contain a bridge between the 2′ and the 5′ positions of the base ring, which locks the oligonucleotide in rigid confirmation and disrupts the 2′ ribose position (Fig. 4*G*). To determine if tDR-GlyGCC-30 harboring LNA bases (instead of a normal hydroxyl group) reduces HDL-sRNA affinity, MST assays were completed. Based on MST, HDL were found to have decreased affinity toward LNA-tDR-GlyGCC-30 compared to the native form of tDR-GlyGCC-30 (Fig. 4*H*). These results support that the 2′ hydroxyl promotes sRNA binding to HDL as disruption of this position through two different approaches both reduced HDL-sRNA binding affinity. Natural sRNAs have phosphodiester bonds (Fig. 4*I*) between nucleosides which are sensitive to RNase hydrolysis. To increase sRNA stability for experimental studies, oligoribonucleotides are often synthesized with phosphorothioate bonds (Fig. 4*I*) to increase resistance to RNases digestion, e.g. siRNAs for gene silencing (45). Based on MST analyses, HDL were found to bind to both phosphodiester and phosphorothioate forms of tDR-GlyGCC-30 with strong affinities (Fig. 4*J*). Collectively, these results demonstrate critical features of sRNA biology that influence HDL association.

### apoA-I is a sRNA-binding protein (Fig. 5)

**Figure 5:**
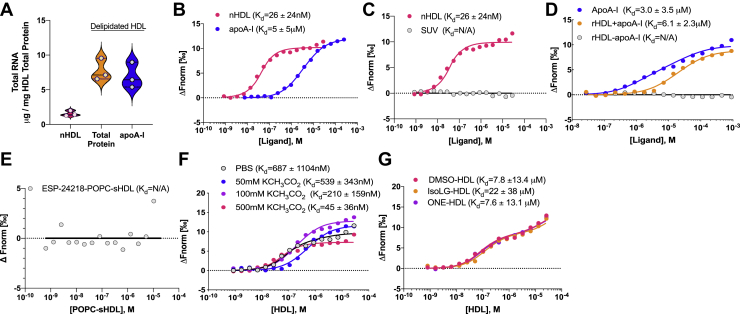
**tDR-GlyGCC binds to apolipoprotein A-I.***A*, total RNA content as determined by direct SYTO RNASelect staining (50 μM) on matched human native DGUC-HDL (nHDL), delipidated nHDL (total protein), and (FPLC) purified apoA-I from delipidated nHDL. Violin plot showing median and quartile ranges, n = 3. *B*, MST analysis of interactions with tDR-GlyGCC-30 and human DGUC-HDL purified apoA-I or nHDL. *C*, MST analysis of interactions with tDR-GlyGCC-30 and small unilamellar vesicles (SUV) or nHDL. *D*, MST binding curves of tDR-GlyGCC-30 binding to purified apoA-I, or reconstituted HDL (rHDL) with or without apoA-I (rHDL ± apoA-I). *E*, MST binding curves of tDR-GlyGCC-30 binding to synthetic apoA-I mimetic peptide (ESP-24218) reconstituted with 1-palmitoyl-2-oleoyl-sn-glycero-3-phosphocholine (POPC) to form ESP-24218-POPC-sHDL. *F*, MST binding curves of tDR-GlyGCC-30 binding to nHDL with increasing concentrations of potassium acetate (KCH_3_CO_2_, 50–500 mM) in the PBS buffer. *G*, MST binding curves of tDR-GlyGCC-30 and nHDL treated with vehicle (DMSO), isoLG (isolevuglandins), or ONE (lipid aldehyde 4-oxo-2-nonenal). Binding affinities (K_d_) of mean MST analysis from triplicate assays are presented, K_d_ are mean ± SD. DGUC, density-gradient ultracentrifugation; HDL, high-density lipoprotein; MST, microscale thermophoresis; tDRs, tRNA-derived sRNAs.

Based on tDR-GlyGCC-30’s strong affinity to HDL, we sought to determine if sRNAs bind to the phospholipid or protein components of the particle. We first assessed if the RNA cargo separates with lipid or protein cargo by delipidation studies. HDL were isolated from human plasma by DGUC and delipidated using the Bligh–Dyer method to separate phospholipids away from protein cargo. Total RNA levels were quantified in input DGUC-HDL and matched delipidated total protein post-Bligh–Dyer lipid extraction by SYTO RNASelect, and the levels of detectable total RNA on remaining HDL samples were not reduced by phospholipid removal (Fig. 5*A*). In addition, apoA-I was further isolated from the delipidated total protein samples by ion exchange separation (on AKTA pure FPLC system) and tested for sRNA content, which showed that apoA-I retained high levels of total RNA after delipidation of the HDL particles and ion exchange separation of apoA-I from other HDL proteins (Fig. 5*A*). These results suggest that sRNAs likely associate with the protein component of HDL, namely apoA-I, as opposed to HDL phospholipids. To determine if apoA-I is a novel RNA binding protein, human apoA-I was purified from DGUC delipidated HDL using ion exchange columns and tested for binding affinity to tDR-GlyGCC-30 with MST. ApoA-I was found to bind to tDR-GlyGCC-30 with moderate affinity, confirming that apoA-I is likely an RNA binding protein (Fig. 5*B*). As a negative control, actin (isolated from rabbit muscle) was observed to have very weak binding to tDR-GlyGCC-30 by MST (Fig. S3*A*). In addition to tDR-GlyGCC-30, apoA-I was also found to bind to tDR-GluCTC-30, miR-223-3p and miR-375-3p (Fig. S3, *B*–*D* and Table S7). To determine if tDR-GlyGCC binds to phospholipids, PC-based small unilamellar vesicles (SUVs) were generated and tested with MST. All sRNAs tested completely failed to bind to phospholipid SUVs, including tDR-GlyGCC-30, tDR-GluCTC-30, miR-223-3p and miR-375-3p (Figs. 5*C* and S3, *E*–*G*). These findings indicate that extracellular sRNAs do not directly bind to phospholipids on lipoproteins. Since sRNAs were observed to bind to native HDL with greater affinity than lipid-free apoA-I (Fig. 5*B*), MST assays were used to quantify sRNA binding to reconstituted HDL (rHDL), and tDR-GlyGCC-30 was observed to have moderate binding affinity to rHDL with apoA-I (rHDL+apoA-I); however, tDR-GlyGCC failed to bind to rHDL-like lipid particles when apoA-I was not present on rHDL (rHDL-apoA-I) (Fig. 5*D*). To determine if sRNAs bind to generic lipoproteins or protein–lipid complexes mimicking HDL, synthetic HDL (sHDL) were generated through reconstitution of synthetic apoA-I mimetic peptides with distinct phospholipids shells. For example, ESP-24218, a synthetic apoA-I mimetic peptide consisting of 22 amino acids (46), was reconstituted with 1-palmitoyl-2-oleoyl-sn-glycero-3-phosphocholine (POPC) to form ESP-24218-POPC-sHDL. Strikingly, ESP-24218-POPC-sHDL failed to bind to tDR-GlyGCC-30 in MST assays (Fig. 5*E*). To test if tDR binds to ESP-24218-sHDL composed of other phospholipid species, sHDL were reconstituted individually using 1,2-dimyristoyl-sn-glycero-3-phosphocholine (DMPC), 1,2-dipalmitoyl-sn-glycero-3-phosphocholine (DPPC), 1,2-distearoyl-sn-glycero-3-phosphocholine (DSPC), 1,2-dipalmitoyl-sn-glycero-3-phosphorylglycerol (DPPG), or 1,2-Dimyristoyl-sn-glycero-3-phosphoethanol-amine (DMPE). Despite altering the phospholipid chain lengths and head groups, sHDL particles were not observed to bind to sRNAs by MST analyses (Fig. S4, *A*–*E*). These results support that apoA-I is a novel RNA binding protein for sRNAs, including tDRs and miRNAs. Moreover, results indicate that apoA-I and not phospholipids confer HDL particle binding and association to extracellular sRNAs.

To further investigate the biochemical properties of HDL-sRNA binding, multiple physicochemical approaches were taken with MST assays. To determine if sRNA binding to HDL is ionic, MST assays were completed with human DGUC-HDL and tDR-GlyGCC-30 in a graded series of increasing potassium acetate concentrations. Remarkably, we found that HDL–sRNA binding affinities were increased with increasing concentrations of potassium acetate (Fig. 5*F*). These results indicate that HDL binding to sRNAs, *e.g.*, tDR-GlyGCC-30, is not likely to be ionic, as the high salt concentrations increased binding affinity and could suggest that the association between HDL and extracellular sRNA is hydrophobic. To further define chemical influences or regulators of HDL-sRNA binding, we sought to determine the impact of reactive modifications on the binding affinity between HDL particles and extracellular sRNAs, including isolevuglandin (IsoLG) and 4-oxo-nonenal (ONE) that modify positively charged residues of apoA-I (*i.e.*, lysines and arginines) that have the potential to form ionic bonds with the negatively charged backbone of sRNAs. In atherosclerotic cardiovascular disease (ASCVD), HDL lose many of its beneficial functions, including cholesterol efflux acceptance capacity and reactive modifications to HDL’s lipids and protein (amino acids) likely confer these functional changes (47, 48). We have previously reported that IsoLG modifications to HDL particles are increased in atherosclerotic cardiovascular disease and associated with decreased cholesterol efflux capacity (49). We have also found that ONE modifications also reduce HDL cholesterol acceptance capacity (50). To determine if IsoLG or ONE modifications also alter HDL’s ability to bind to sRNAs, DGUC-HDL was treated with 1 M equivalents of IsoLG, ONE, or dimethylsulfoxide (DMSO) (vehicle control), and modified HDL were tested for binding with MST. Although these modifications alter HDL’s ability to accept cholesterol and other functions, they did not alter HDL’s ability to bind to tDR-GlyGCC-30 (Fig. 5*G*), further supporting the notion that the association between HDL and extracellular sRNA is hydrophobic.

## Discussion

HDL transport many different types of molecules; however, the mechanisms by which HDL interacts with nonlipid cargo are unknown. The purpose of this study was to characterize the biochemical interactions between HDL and sRNA. Here, we demonstrate that HDL binds to extracellular sRNAs potentially through apoA-I, for which RNA binding is a previously unrecognized biological function. Results indicate that HDL carry high concentrations of tDRs, including tDR-GlyGCC-30. This tDR was highly abundant on HDL and was found to be significantly increased in subjects with coronary atheroma as determined by the presence of raised calcified lesions by non-contrast cardiac computed tomography (CT) (CAC>0). Macrophages may contribute to the HDL-tDR signature as HDL were found to readily accept tDRs from primary macrophages. HDL-sRNA binding assays were completed by MST and confirmed by EMSA. These assays showed that HDL have strong affinity toward extracellular sRNAs, including tDRs and miRNAs, with dissociation constants (K_d_) in the nM range. To define the physicochemical properties that regulate and influence HDL–sRNAs, tDR-GlyGG-30 was synthesized in multiple chemical forms, lengths, structures, and modifications to test in HDL binding assays. In this study, we found that the 2′ hydroxyl group present on nucleotide ribose is a strong determinant of HDL binding. For example, DNA (lacking the 2′-hydroxyl group), 2′-*O*-methylation of sRNA, and locked confirmation of the 2′ position were each found to disrupt HDL-tDR binding. In subsequent experiments, we sought to determine the impact of specific reaction conditions on HDL-sRNA binding as a means to further understand the biochemistry of the association. We observed that high-salt conditions greatly increased HDL affinity toward tDR-GlyGCC-30, suggesting that the interaction between apoA-I and tDRs may not be solely through electrostatic interactions and may be conferred by hydrophobic interactions.

Results showed that human DGUC-HDL transports high concentrations of extracellular sRNAs in circulation, and overall sRNA levels were correlated to TC levels in both control and atherosclerosis subjects (CAC). The use of SYTO RNASelect has allowed for the determination of total RNA content on HDL particles without the need to isolate RNA or have prior knowledge of candidate sRNAs for PCR quantification. Based on a ssRNA standard curve, we demonstrated that HDL transports approximately 125 μg of RNA per mg of HDL total protein. This level of RNA is much greater than previously predicted or calculated based on quantitative PCR of individual miRNAs (26, 51, 52). Based on sRNA-seq results, we also now recognize that host miRNAs are only a small percentage of the total amount of sRNAs on circulating HDL. The functional impact of HDL-sRNAs may not be restricted to a few select miRNAs, but most likely is conferred by the multitude of host and non-host sRNA classes, namely tDRs and rDRs, that make up the majority of sRNAs on HDL. Conventional sRNA-seq approaches rely on a 5′ terminal nucleotide monophosphate and 3′ terminal nucleotide hydroxyl group for adapter ligation strategies. These required terminal chemistries are often produced by RNase III enzyme-mediated cleavage of the sRNA fragment from a longer parent transcript. Parent tRNAs have the potential to be cleaved by non-RNase III enzymes that fail to leave the required chemistries for sRNA-seq. For example, tDRs can harbor terminal 2′,3′- cyclic phosphate chemistries due to intermediate cleavage by endoribonucleases (*e.g.*, with RNase A, Rnase T1, and Rnase T2) or final cleavage by endonucleases (*e.g.*, ANG, IreI, PP11, and USB1), resulting in them escaping 3′-adapter ligation and not being captured by sequencing (53). Recent advances in methodology highlight ways to overcome these hurdles including removal of all terminal chemistries and chemical additions to create required chemistries through, *e.g.*, enzymatic treatment with T4 polynucleotide kinase to generate 5′ terminal monophosphate (54). Moreover, posttranscriptional modifications, *e.g.*, m^1^A and m^1^G base methylations, have been reported to block reverse transcription and cDNA library preparation for modified sRNAs. Recent improvements in demethylase-based sRNA-seq have emerged as tools to overcome these potential barriers. For example, demethylation with α-ketoglutarate-dependent hydroxylase prior to sRNA-seq has been reported to increase tDR capture in sRNA-seq datasets (55). One limitation of these study is that we applied conventional sRNA-seq approaches to quantify HDL-tDRs. We predict that if demethylase steps were applied prior to sRNA-seq and if we addressed potential sRNA terminal chemistries issues, we would observe even more tDRs on circulating HDL (56). While these method advances do not overcome all terminal chemistry issues or posttranscriptional modifications (56, 57), future studies employing these methodologies may help to better capture the full profile of HDL-sRNA profile. Nonetheless, several tDRs were found altered in this study, and unmodified tDR-GlyGCC was found to have high affinity to HDL and exported from macrophages.

The most important advance of this study is the observation that HDL binds to sRNAs through protein (*e.g.*, apoA-I) interactions, not phospholipids. We have previously predicted that extracellular sRNAs may bind to HDL through binding to zwitterionic lipids (PC) within the phospholipid shell of HDL particles (8). Oligonucleotides have been previously reported to bind to and associate with zwitterionic lipids, potentially through a divalent cation bridge between the positively charged choline headgroup on PC and the negatively charged backbone of RNA (23, 58, 59). Others have expanded on this discussion toward a model in which an unknown RNA binding protein(s) on HDL, as opposed to phospholipids, confer the association of sRNAs to HDL particles (26). In agreement with data from Plochberger *et al.*, our study clearly showed that phospholipid vesicles and/or particles did not bind to the sRNAs we tested, whereas apoA-I protein did bind to multiple sRNAs. In this study, multiple approaches were used to determine if sRNAs bind to the protein or lipid component of HDL, and all evidence from this study supports that it is indeed a protein, not lipid, that mediates the association of sRNAs to HDL particles. For example, removing phospholipids from HDL particles did not change the amount of detectable RNA on HDL particles. Moreover, apoA-I, which accounts for approximately 70% of the protein mass on HDL, was demonstrated to be a novel RNA binding protein and bind to tDR-GlyGCC with moderate affinity, slightly less than HDL particles which showed strong affinity. tDR-GlyGCC-30 and other sRNAs were also found to bind to rHDL; however, construction of rHDL-like particles without apoA-I failed to produce particles that would bind to extracellular sRNAs. Collectively, these strongly suggest that sRNAs bind to the protein component, specifically apoA-I, and not the phospholipid component of HDL, as we have previously hypothesized (8). Although apoA-I accounts for approximately 70% of HDL total protein, the pool of HDL particles transport many proteins. Therefore, it is unlikely that apoA-I is the only protein on HDL that has RNA binding properties. This is also reflected in the results which showed that HDL particles bind to sRNAs with higher affinity than lipid-poor apoA-I or rHDL generated from recombinant apoA-I.

Differential expression analysis identified many significantly altered tDRs on HDL in atherogenic subjects, including tDR-GlyGCC-30. The detection of tDR-GlyGCC in our HDL sequencing dataset is not uncommon, as this sequence has been detected in many biological samples, including both cellular and extracellular RNA samples (28, 29, 32, 40, 42, 60). The underlying reason for tDR-GlyGCC high abundance in plasma may be related to increased stability and resistance to RNase digestion, as opposed to increased synthesis and secretion from donor cells (40, 41). tDR-GlyGCC is likely detected in most extracellular sRNA-seq datasets due to its potential resistance to plasma RNases that degrade other secreted tDRs. For instance, tDR-GlyGCC may harbor various RNA modifications that do not perturb sequencing or qPCR and that may contribute to its increased stability in circulation (61). Such modifications that help to stabilize tDRs/tRNAs include pseudouridylation, which is found to improve tDR binding affinity (62) and Dnmt2-mediated methylation (m^5^C), which protects against heat shock (63) and alters tDR secondary structure that was resistant to RNases (64). Its protection from digestion may also be related to its high potential to form homodimers with other tDR-GlyGCC or even heterodimers with tDR-GluCTC (40, 41). Furthermore, tDR-GlyGCC may even form a small 6 nt hairpin on its 5′ terminal end that may contribute to increased stability. For MST testing, all oligos were denatured with heat prior to analyses to prevent secondary structures; however, no differences were observed with tDR-GlyGCC-30 binding to HDL with or without heat denaturing prior to MST analysis (data not shown). We did observe, however, that HDL displayed preferential binding to ssRNA over dsRNA forms of tDR-GlyGCC, and HDL binding affinity to dsRNA-tDR-GlyGCC was restored when dsRNA-tDR-GlyGCC-30 was denatured (by heat) prior to MST analysis. These results support that while tDR-GlyGCC’s potential to form self-hairpins and/or homodimers may increase their stability in biofluids and increase the chance of associating lipoproteins, HDL’s affinity to tDR-GlyGCC is not likely affected by this potential secondary structure.

The biological functions of tDRs on HDL are unknown, but cellular tDRs have emerged as key regulators of cellular stress and protein synthesis (29, 33). HDLs have the capacity to deliver functional miRNAs to recipient cells, including endothelial cells (9, 11, 12); however, HDL-tDR transfer to endothelial cells or other recipient cells has not been demonstrated to date. It is plausible that HDL does transfer tDRs, like miRNAs, to recipient cells and regulate gene expression, but these studies will require further investigation in the future. Currently, it is unknown if HDL can transfer sRNAs to immune cells; however, if HDL is taken up by immune cell phagocytosis or fluid-phase uptake, then HDL-tDRs cargo may activate ssRNA sensing Toll-like receptors 7/8 (TLR7/8) in immune cells, leading to proinflammatory gene expression, cytokine secretion, and inflammation. HDL is generally anti-inflammatory in health but may promote inflammation in certain diseases or conditions (65). HDL’s ability to bind and sequester extracellular tDRs, particularly nonhost tDRs, likely serves as an anti-inflammatory function to prevent excess activation of pattern recognition receptors, *i.e.*, TLR7/8, in immune cells. HDL also may regulate genes through acceptance of regulatory sRNAs away from donor cells. Here, we show that macrophages export tDRs to HDL; however, future studies are needed to define the impact of tDR export on macrophage gene expression. Likewise, future investigation is needed to determine if macrophages export tDRs to HDL in response to specific cellular stresses. Although macrophages export tDRs to HDL, the mechanism by which HDL acquires tDRs from macrophages is currently unknown. It is possible that macrophages and other cell types simply export unprotected and free sRNAs from the cell, and HDL have the capacity to bind and protect free sRNAs. In this model, HDL would interact with extracellular sRNAs independent of cells and would be entirely dependent on HDL’s affinity for sRNA. To calculate HDL’s binding affinity (K_d_, dissociation constant) to sRNAs and determine the underlying biochemistry of their association, MST and EMSA assay were used. Remarkably, HDL were found to have very strong binding affinity toward ssRNA forms of tDR-GlyGCC-30. While these assays were performed with synthetic oligos and various modifications were tested, future studies are warranted to employ methods that enable purification of *in vivo* endogenously modified tDRs such as using anion exchange columns with affinity capture (66) or pulldown with biotinylated complementary DNA probes (67) and compare these to the synthetic tDR binding. The clinical applicability of these findings are unknown; however, therapeutic strategies based on HDL’s ability to bind to and delver RNAs to specific cells may ultimately elevate HDL as a potential RNA-based drug delivery vehicle. Conversely, results presented here may allude to a phenomenon in which HDL readily binds to extracellular sRNAs to sequester potentially inflammatory signals away from pattern recognition receptors. Future work is needed to define the role of HDL in dampening uncontrolled immune responses due to extracellular sRNAs; however, if established then HDL-based therapies may be applied to sepsis and infection.

Collectively, results support that HDL transport high concentrations of extracellular sRNAs, including both host and nonhost tDRs. HDL was determined to bind to tDRs through the novel RNA binding protein apoA-I which also serves as the structure-function protein for HDL particles. HDL was found to bind to RNA over DNA and single-stranded over double-stranded forms. Key biochemical experiments showed that a hydroxyl group at the 2′ position of the ribose was determinative toward HDL binding and disruption of this group reduced HDL binding affinity to sRNAs. Likewise, HDL association to sRNAs might be mediated through hydrophobic interactions as HDL-sRNA binding was greatly increased in a high salt buffer. Overall, results from our investigation of the underlying biochemistry of HDL’s association to sRNAs revealed key fundamental principles of HDL’s transport of tDRs in ASCVD and HDL’s affinity toward extracellular sRNAs. These results will serve as a platform for future studies to manipulate HDL-sRNA cargo for therapeutic approaches to prevent or treat ASCVD.

## Experimental procedures

### Reagents

Synthesized oligonucleotides used in this study are listed in Supporting Information Table S7.

### Clinical assays

Plasma was collected from whole venous blood (K_2_EDTA) obtained from participant donors following informed written consent. Experiments were performed under institutional review board approved protocols from Vanderbilt University Medical Center (IRB#170046 and #101615). Participants underwent noncontrast coronary CT examination to identify the presence of calcified coronary atheroma and measure the CAC score (68, 69). Women who were pregnant or potentially pregnant were excluded from the CT examination. Image acquisition was performed using a 64-slice multi-detector CT scanner (Brilliance 64, Philips Healthcare). Technical parameters included 120 KVp, 150 mAs, and ECG-gating of image acquisition in late diastole. CAC was measured on 3.0 mm thick slices using the Food and Drug Administration–approved calcium scoring software (Philips IntelliSpace Portal, Philips Healthcare) and reported as the Agatston score (70) for minimum lesion volume of 0.5 mm3 and an attenuation threshold of ≥130 Hounsfield units. Subject characteristics are presented in Table S1. Plasma lipoprotein cholesterol and plasma triglyceride levels were quantified using an Ace Axcel clinical chemistry system (Alfa Wassermann). These studies abide by the Declaration of Helsinki principles.

### Lipoprotein isolation

DGUC was completed, as previously reported (71). Native human HDL (1.063–1.021 g/L) were isolated by sequential DGUC using an Optima XPN-80 Ultracentrifuge with SW32Ti or SW41Ti rotors (Beckman-Coulter). Lipoproteins were dialyzed in 1× PBS (4L) for at least four bucket changes and concentrated with 3 kDa m.w. cutoff filters (Millipore). Isolated human DGUC-HDL, cell culture media, or plasma samples were injected into an AKTA pure FPLC system (Cytiva) with three tandem Superdex-200 Increase SEC columns (Cytiva) and collected in 1.5 ml fractions in running buffer (10 mM Tris-HCl, 0.15 M NaCl, 0.2% NaN_3_), as previously described(21). SEC fraction fluorescence was measured with microplate fluorometer (SynergyMx, Biotek). DGUC-HDL or individual SEC fractions were assessed for total protein (Pierce BCA, ThermoFisher), TC, PC, and triglyceride (Pointe Scientific) by colorimetric assays.

### HDL delipidation and apoA-I purification

DGUC-HDLs were delipidated as previously described (9). Briefly, 6 ml of ice-cold methanol:chloroform (1:1) was added to 1 ml of lyophilized DGUC-HDL, mixed, and incubated on ice for 30 min. Chilled methanol (4 ml) was added to the mixture, centrifuged (3000 rpm) for 10 min, removed supernatants, and repeated until pellet turned white. Chilled methanol (10 ml) was re-added, mixed, and recentrifuged. The pellet was dried under nitrogen, resolubilized with 6M guanidine HCl, and dialyzed in ammonium bicarbonate (10 mM) overnight. ApoA-I was isolated from delipidated HDL with 6M Urea by anion exchange chromatography with Q-Sepharose Fast Flow column coupled with a Q-Sepharose High-Performance column on an AKTA pure FPLC system (Cytiva). ApoA-I fractions were confirmed using Coomassie-based Aquastain (BulldogBio) on an SDS-PAGE gel and were dialyzed in ammonium bicarbonate (10 mM).

### HDL reconstitution

rHDL was prepared with DGUC-HDL purified apoA-I and L-α-PC (Avanti Polar Lipids) at 100:1 (PC:Protein). Lyophilized PC was resuspended in PBS (80 μl:1 mg PC) and vortexed. 10% sodium deoxycholate was added (1/5 total volume), vortexed, and incubated at 37 °C for 30 min in water bath. Purified apoA-I was added to the reaction and was incubated at 37 °C for 1 h. Samples were dialyzed, and rHDL were assessed by SEC. As a control, SUVs were prepared using evaporated Egg PC (Avanti Polar Lipids) and Hepes Buffered Solution (20 mM Hepes, 150 mM NaCl) and incubated for 1 h RT. Samples were vortexed vigorously, sonicated, and prepared SUVs were analyzed by SEC and stored at 4 °C.

### Preparation of sHDLs

22A peptide (PVLDLFRELLNELLEALKQKLK) was synthesized by Genscript. POPC, 1,2-dimyristoyl-sn-glycero-3-phosphocholine, DMPC, 1,2-dipalmitoyl-sn-glycero-3-, DSPC, 1,2-distearoyl-sn-glycero-3-phosphocholine, DPPG-Na, 1,2-dipalmitoyl-sn-glycero-3-phosphorylglycerol sodium salt, and DMPE, 1,2-dimyristoyl-sn-glycero-3-phosphoethanol-amine were purchased from NOF America Corporation. 22A peptide and phospholipids were dissolved in acetic acid and mixed at a peptide/lipid mass ratio of 1:2 and lyophilized overnight. The lyophilized powder was rehydrated with PBS (pH 7.4). The solution was then cycled three times between 50 °C (10 min) and room temperature (10 min) to allow the formation of sHDL complexes. The hydrodynamic size of the resulting sHDLs was analyzed by dynamic light scattering using Zetasizer Nano ZSP (Malvern Instruments). The purity of sHDLs was analyzed by gel permeation chromatography using a Tosoh TSK gel G3000SWxl column (Tosoh Bioscience) with UV detection at 220 nm.

### HDL dicarbonyl modifications

To modify HDL with reactive dicarbonyls modifications, DGUC-HDL were reacted with 1 M equivalence (eq) IsoLG to HDL-apoA-I protein levels, 1 M eq of ONE, or control vehicle DMSO. The reaction was completed overnight at 37 °C.

### Microscale thermophoresis

Synthesized AF647-labeled oligoribonucleotides (100 nM, IDT, Table S7), denatured at 90 °C for 2 min and then cooled, were incubated (1:1) with 16 serial dilutions of ligand (*e.g.* HDL) in PBS at RT for 5 min protected from light. sRNA-ligand solutions were loaded into standard-treated glass capillaries (NanoTemper Technologies) and tested using a Monolith NT.115 instrument (NanoTemper Technologies) at the Vanderbilt Center for Structural Biology Instrumentation Core. Other buffers also include PBS with potassium acetate (Research Products International). For all MST experiments, 20% excitation power and 40% MST power were used. Each capillary was scanned to assess fluorescence homogeneity and fluorescence adsorption to ensure adequate sample quality, and MST measurements were then performed using the MO.Control software (v1.6, NanoTemper Technologies). Data analyses of repeated measurements were calculated with the MO.Affinity Analysis software (v2.3, NanoTemper Technologies), and representative binding curves were presented in figures. The dissociation constant (K_d_) was determined by the MO.Affinity Analysis software. Interassay variability was noted between HDL donors; therefore, mean traces of MST curves are presented along with K_d_ ± SD. This observed variability is likely due to population differences in HDL molecular weight, which we kept constant at an assumed mass of 360 kDa.

### Electrophoretic mobility shift assays

EMSA were performed as previously described (44). Briefly, AF546-tDR-GlyGCC-30 (1 μM) was incubated with HDL (serial dilutions) and crosslinked (UV 254 nm) for 15 min on ice. Cross-linked samples were separated by native gel electrophoresis—NativePAGE 4 to 16% gel (ThermoFisher) with 4× NativePAGE sample buffer (ThermoFisher) and 0.5× TBE running buffer. Fluorescence signal (representing migration of tDR-GlyGCC-30) in the gel was visualized with Green Epi Illumination and 602/50 emission filter using a Digital ChemiDoc MP (Bio-Rad), and protein staining was completed by Coomassie-based Aquastain (Bulldog Bio). Sample protein masses were assessed with Amersham high molecular weight protein marker mix (GE Healthcare) with proteins porcine thyroglobulin (R_s_ = 17.0 nm), equine spleen ferritin (R_s_ = 12.2 nm), bovine heart lactate dehydrogenase (R_s_ = 8.2 nm), and bovine serum albumin (R_s_ = 7.1 nm) (50).

### Animals

WT C57BL/6 mice from The Jackson Laboratory were used under approved Vanderbilt Institutional Animal Care and Usage Committee protocols. Mice were housed in a 12 h light/dark cycle with unrestricted access to standard chow (NIH-31) and water. Male 8 to 10-week-old mice were used in this study.

### Tissue culture

Human PBMCs were isolated by Ficoll-gradient centrifugation, washed, and counted. PBMCs were incubated with anti-human CD14 MicroBeads (Miltenyi Biotec), and monocytes were separated on a LS magnetic column on a QuadroMACS separator (Miltenyi Biotec). Cells were differentiated with human GM-CSF (50 ng/ml, Sino Biological) in RPMI 1640 medium with L-glutamine (300 mg/L, ThermoFisher), supplemented with 10% heat-inactivated fetal bovine serum (FBS, ThermoFisher) and 1% penicillin/streptomycin (Gibco) for 7 days followed by 24 h of native HDL (1 mg/ml) treatments in FBS-free media. Bone marrow cells were isolated from WT C57BL/6 mouse femurs and differentiated to BMDMs using murine GM-CSF (50 ng/ml, Tonbo Biosciences) in Dulbecco's modified Eagle's medium (ThermoFisher) supplemented with sodium pyruvate (110 mg/L), L-glutamine (584 mg/L), 10% heat-inactivated FBS (ThermoFisher), and 1% penicillin/streptomycin (Gibco) for 7 days. BMDMs were transiently transfected with tDR-GlyGCC-30-AF647 (1 μg/ml) complexed to DOTAP transfection reagent (Roche) for 24 h in serum-free media followed by native HDL (1 mg/ml) treatments in FBS-free media for 24 h.

### Protein assays

For Western blotting, samples were denatured in 4× sample buffer (Li-Cor Biosciences) with 10% β-mercaptoethanol at 70 °C for 10 min and separated by gel electrophoresis (NuPAGE 4–12% Bis-Tris gel with 1× MES running buffer, ThermoFisher). Gels were transferred to nitrocellulose membranes (ThermoFisher), blocked in Odyssey blocking buffer (TBS, Li-Cor Biosciences) for 30 min, and incubated overnight with rocking at 4 °C with anti-human apoA-I primary antibody (mouse monoclonal, 1:8000, Meridian Life Sciences). Membranes were washed 3× for 10 min with 1× TBS-T (0.1% Tween 20) and incubated with anti-mouse secondary antibody conjugated to IRDye 800CW (Li-Cor Biosciences) for 1 h. Membranes were washed (3 × 10 min) and imaged with the LI-COR Odyssey Infrared Imaging system and quantified using Image Studio Lite and ImageJ software suites. Precision Plus Kaleidoscope Pre-Stained Protein Standards were used for ladder (Bio-Rad). For ELISA, human Apolioprotein A1 ELISA^PRO^ kits (Mabtech) were used to quantify apoA-I levels (as normalized to total protein) on HDL from CAC and healthy donors.

### RNA assays

DGUC-HDL isolated from CAC and healthy donors were assayed for total RNA content using SYTO RNASelect stain (ThermoFisher). DGUC-HDL was incubated with 50 μM of SYTO stain, rocked at 37 °C at 225 RPM for 25 min, and quantified by fluorometry (490/530 nm). HDL total RNA levels were determined using a standard curve of single-stranded oligoribonucleotide (Table S7), and total RNA levels were normalized to total protein levels. Total RNA were isolated from equivalent amounts of HDL total protein using the miRNeasy Mini Kit (Qiagen). For sRNA analyses, RNA were reverse transcribed using the miRCURY LNA RT kit (Qiagen), and qPCR was performed using custom primers (Table S7) and PowerUp SYBR Green 2× Master Mix (ThermoFisher) on QuantStudio6 or 12k Real-Time PCR instruments (ThermoFisher). Relative quantitative values were determined using an arbitrary Ct of 32 (Relative quantitative value = 2^−ΔCTArb^).

### sRNA sequencing

HDL–sRNA libraries were generated using the NEXTFlex Small RNA-Seq Kit v3 with 1:4 adapter dilution and 22 PCR cycles (PerkinElmer). Following PCR amplification, individual libraries were size-selected (136–200 bp) using Pippin-Prep (Sage Science) by 3% agarose gel cassette with Marker P dye (Sage Science). Libraries (cDNA) were quantified by Qubit (High-sensitivity DNA assay kit, ThermoFisher) and checked for quality by bioanalyzer (High-sensitivity DNA chips, 2100 Bioanalyzer, Agilent) prior to multiplexing using equal molar concentrations and concentrated (DNA Clean and Concentrator kit, Zymo) for multiplex sequencing on the NextSeq500 (Illumina) by the Vanderbilt Technologies for Advanced Genomics (VANTAGE) core (Vanderbilt University Medical Center). Samples were demultiplexed and analyzed using the TIGER pipeline (7). Data analyses were completed using the TIGER sRNA pipeline, as previously described.

### Statistics

To determine differential expression (abundance) of HDL–sRNAs by sRNA-seq, DESeq2 analyses were applied as part of the TIGER pipeline (72). For comparisons between two independent groups with n > 3 in each group, nonparametric Mann–Whitney tests were used. For comparisons between two independent groups with n = 3 in each group, unpaired two-tailed *t* tests were used. For correlation analyses, Pearson correlations were used. A *p*-value <0.05 was considered significant. GraphPad Prism 9 (GraphPad Software) was used to generate graphs. Affinity Designer was used to assemble the images and generate the figures.

## Data availability

Sequencing data can be accessed in the publicly available database—Gene Expression Omnibus (GSE191337). Sequencing data will also be made available upon request.

## Supporting information

This article contains supporting information.

## Conflict of interest

The authors declare that they have no conflicts of interest with the contents of this article.
