# Supplementary material for: Elucidation of physico-chemical principles of high-density lipoprotein–small RNA binding interactions
Source: J Biol Chem. 2022 Apr 18;298(6):101952. doi: 10.1016/j.jbc.2022.101952 (PMC9133651; doi:10.1016/j.jbc.2022.101952)
Supplement: Supporting information [file mmc1.pdf]

SUPPORTING INFORMATION

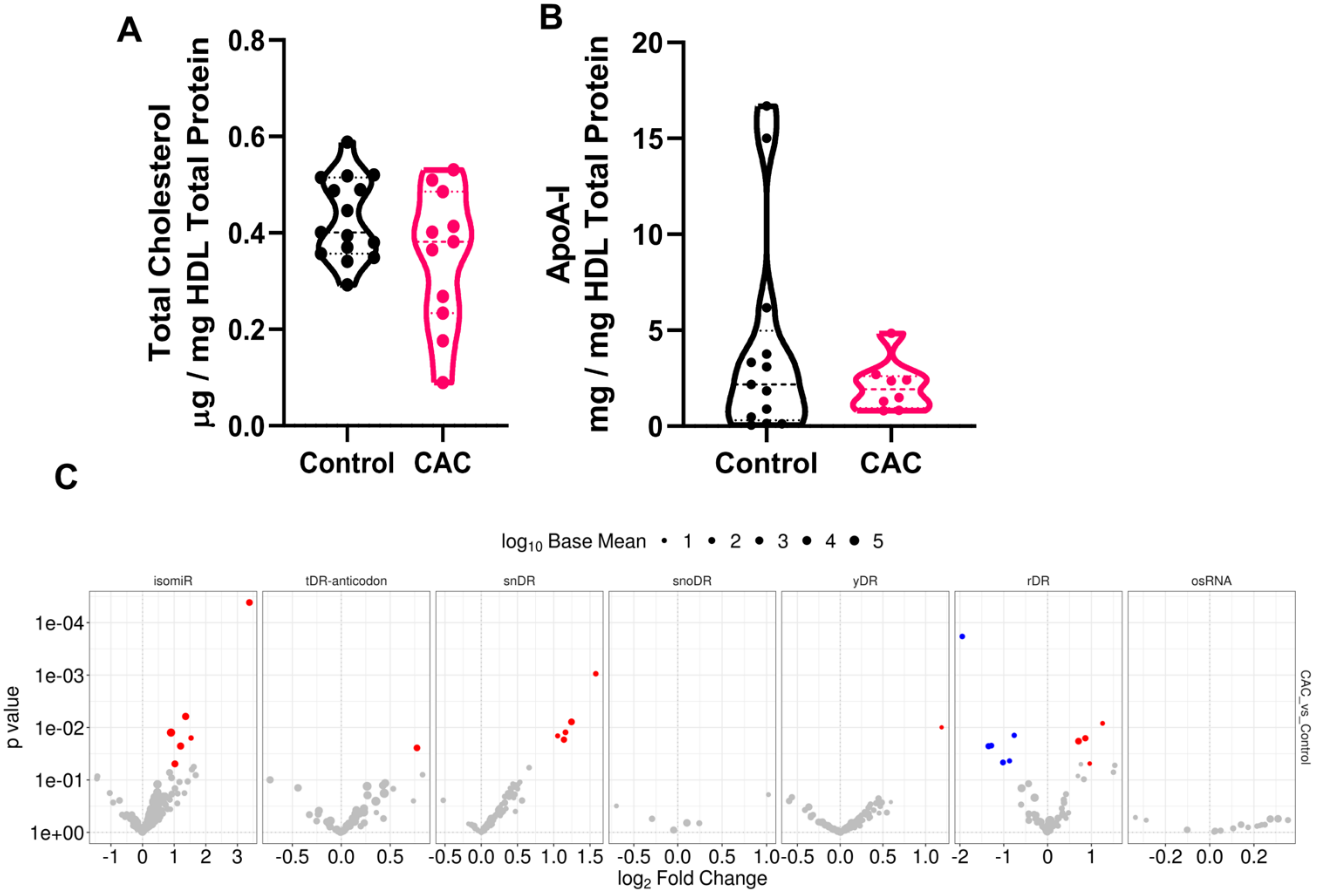

Figure S1. HDL isolated by density-gradient ultracentrifugation (DGUC) was assayed for **A)** total cholesterol and protein by colorimetric kit and **B)** ApoA-I protein by ELISA (n=11 Control, n=11 CAC sujects). Violin plots showing median and quartile ranges. **C)** Differential expression analysis at the parent level from sRNA sequencing of HDL from Control (n=46) and CAC (n=21) Subjects. Volcano plots demonstrating significant DESeq2 analysis and the $\log_2$ fold change. Red, increased; blue, decreased.

A.

B.

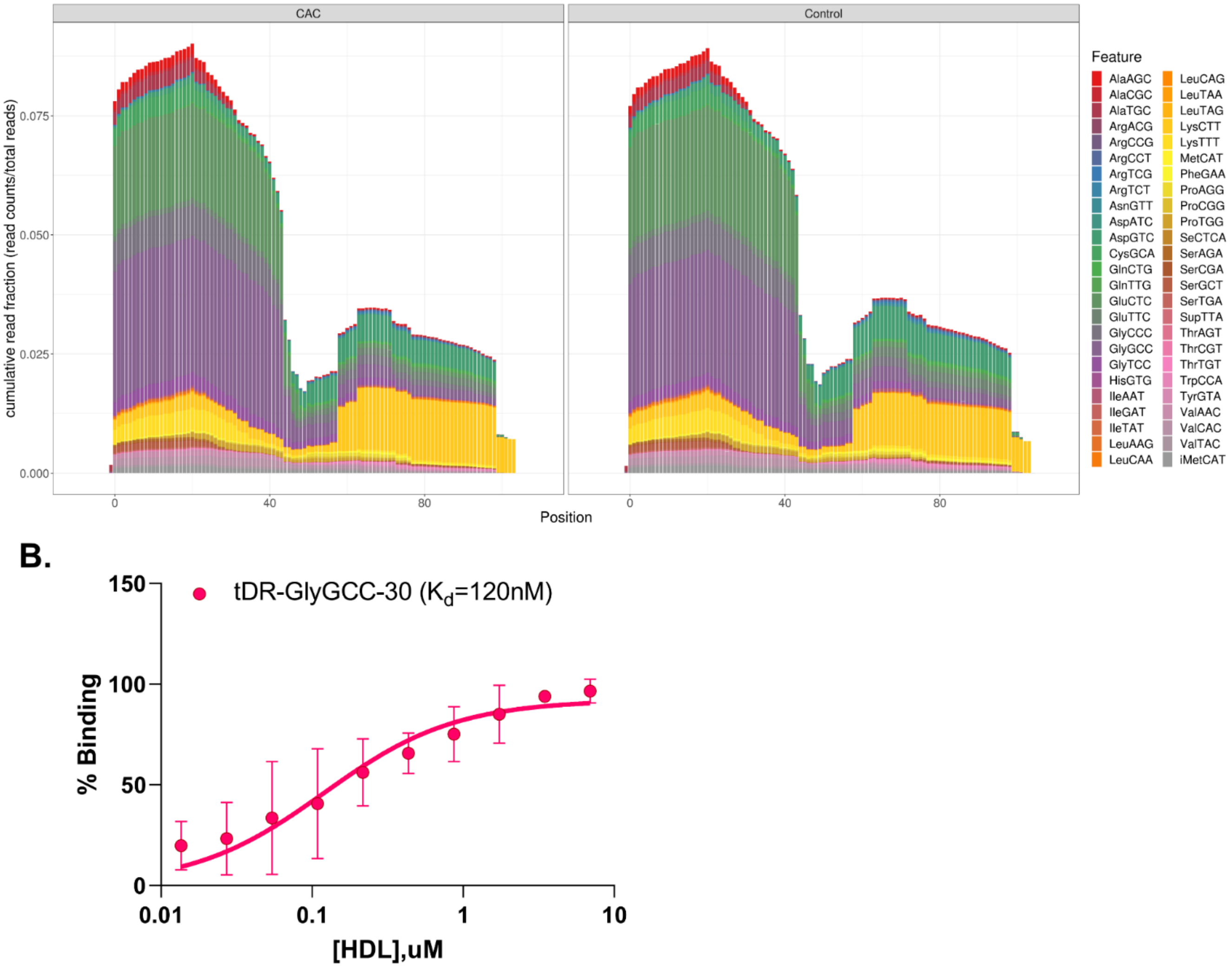

Figure S2. A) Positional coverage of maps of HDL tDRs for parent tRNA amino acid anti-codons, reported as mean cumulative read fraction (read counts/total counts) between Control (n=46) and CAC (n=21) subjects. **B)** Fluorescence electrophoretic mobility shift assay nonlinear regression binding percentage of DGUC-HDL complexed with tDR-GlyGCC-30 labelled with Alexa Fluor 546. Binding affinity (K_d) is shown. n=3, data are mean ± SD.

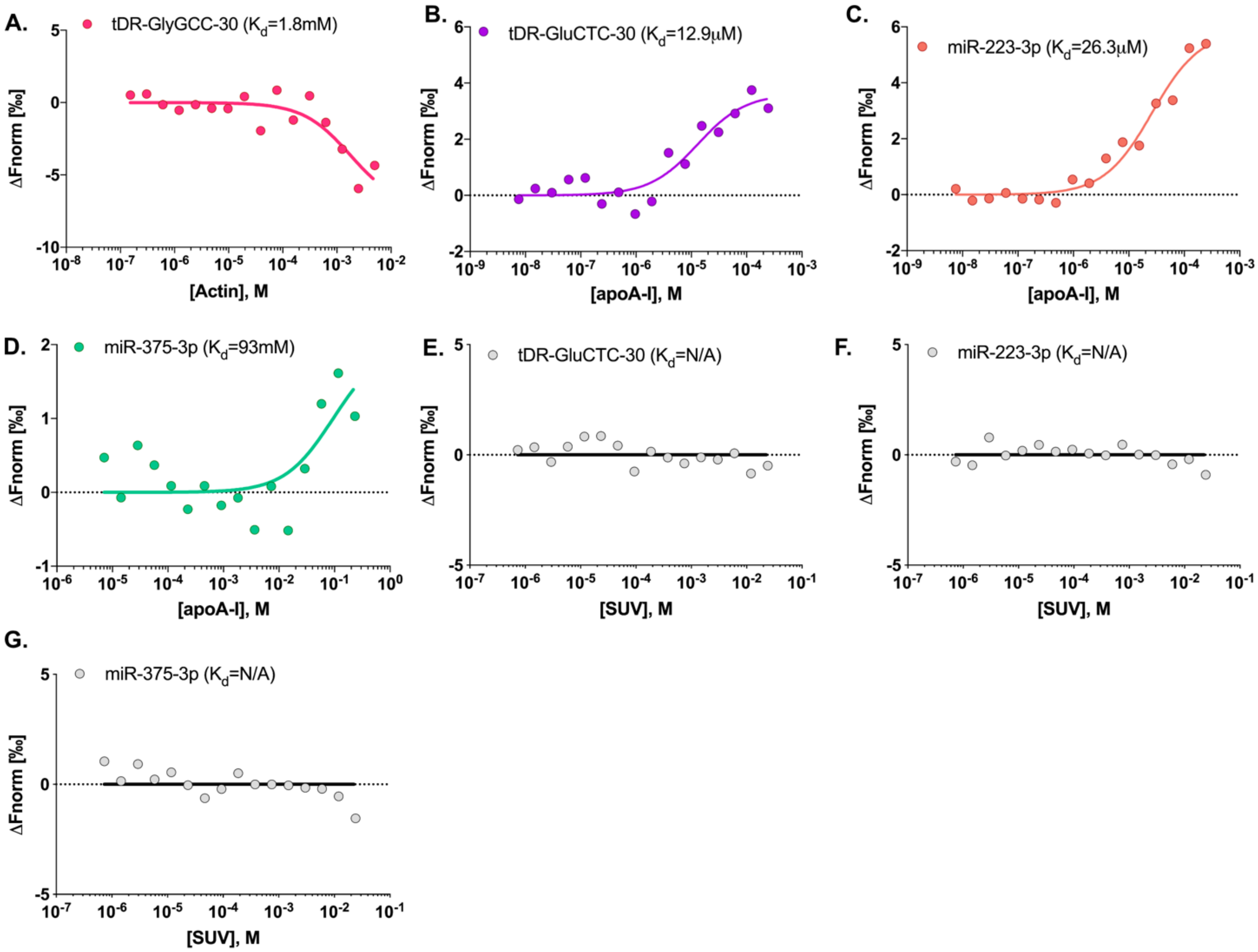

Figure S3. MST analysis of interactions between **A)** tDR-GlyGCC-30 and actin (rabbit muscle, Sigma), apoA-I and **B)** tDR-GluCTC-30, **C)** miR-223-3p, and **D)** miR-375-3p; and single unilamellar vesicles (SUV) and **E)** tDR-GluCTC-30, **F)** miR-223-3p, **G)** miR-375-3p. Binding affinities (K_d) of representative curves are shown.

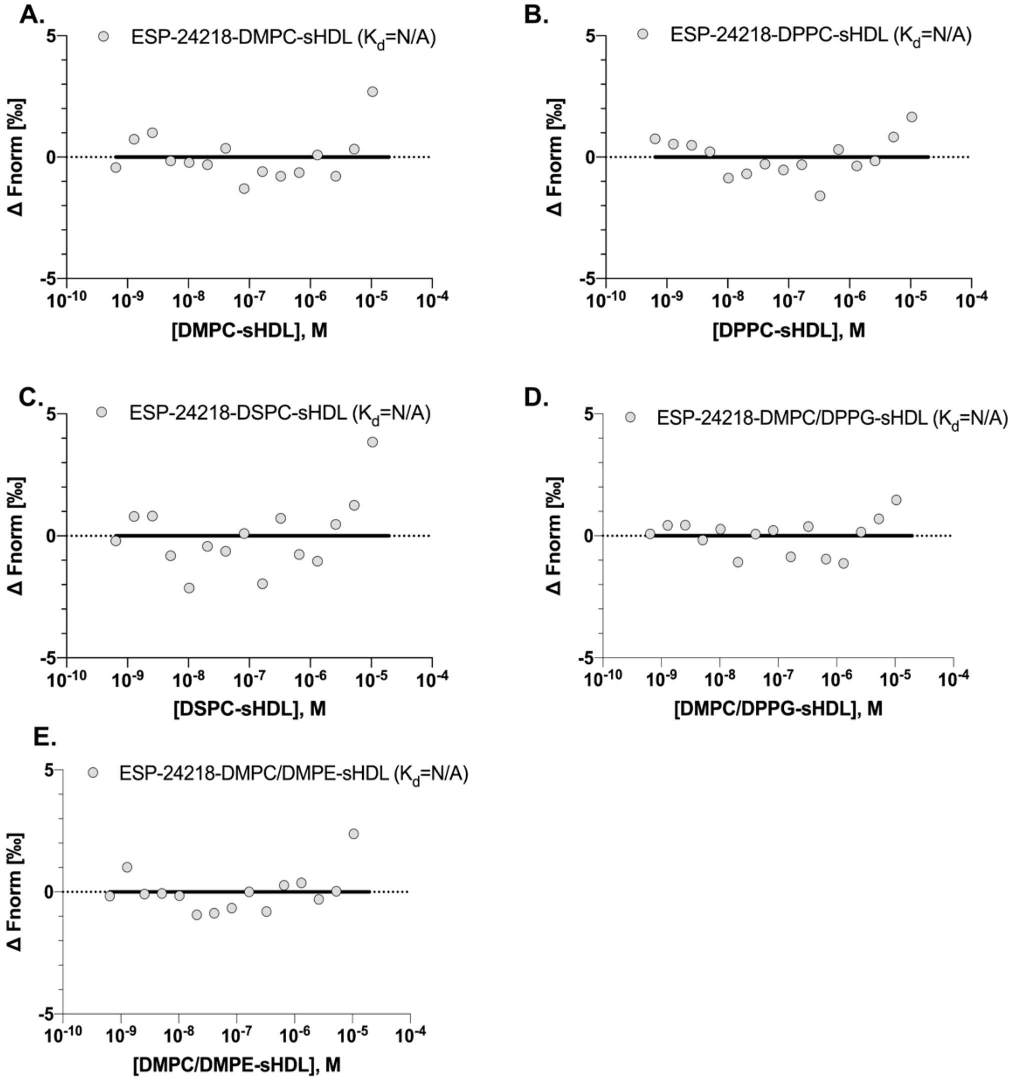

Figure S4. MST analysis of interactions between tDR-GlyGCC-30 and synthetic HDL (sHDL) with apoA-I mimetic peptide, ESP-24218, and either **A)** 1,2-dimyristoyl-sn-glycero-3-phosphocholine (DMPC), **B)** 1,2-dipalmitoyl-sn-glycero-3-phosphocholine (DPPC), **C)** 1,2-distearoyl-sn-glycero-3-phosphocholine (DSPC), **D)** DMPC and 1,2-dipalmitoyl-sn-glycero-3-phosphorylglycerol (DPPG), and **E)** DMPC and 1,2-Dimyristoyl-sn-glycero-3-phosphoethanol-amine (DMPE). Representative curves and binding affinities (K_d) are shown.

Table S1: Clinical parameters for CAC and Control Subjects

	CAC (N=21)	Control (N=46)	P-value
Age, years	61 (54, 67)	45 (37, 56)	<0.001
Race, # (% Caucasian)	18 (86)	39 (85)	0.92
Sex, # (% female)	9 (43)	30 (65)	0.09
Total-C, mg/dL	218 (185, 326)	191 (176, 267)	0.14
HDL-C, mg/dL	53 (49, 74)	57 (51, 67)	0.91
LDL-C, mg/dL	142 (110, 221)	118 (96, 182)	0.23
Triglycerides, mg/dL	103 (73, 171)	90 (76, 117)	0.35
Coronary calcium score, Agatston units	61 (9, 221)	0 (0, 0)	<0.001

Race, Sex, P value Pearson Chi-Square; P value Mann-Whitney U; Median (25% and 75% quartiles)

Table S2. HDL-tDR changes associated with atherosclerosis, as reported by parent tRNA summary counts.

tRNA	pvalue	FoldChange (CAC/Ctr)	Abundance (baseMean)
ThrCGT	2.45E-02	1.71	66.46
MetCAT	9.94E-02	0.60	190.22
GlyTCC	1.15E-01	1.36	1607.17
ValAAC	1.27E-01	1.34	660.84
LysCTT	1.32E-01	1.20	7326.00
AsnGTT	1.41E-01	0.74	312.79
IleGAT	1.48E-01	1.45	18.80
GlyGCC	1.80E-01	1.36	16333.19
AlaTGC	1.96E-01	1.20	1482.58
SerACT	2.48E-01	1.27	11.38
TyrATA	2.51E-01	1.67	9.80

Table S3. Significantly altered host HDL-tDRs in human atherosclerosis (read level).

tDR	Length	P-value	FoldChange (CAC/Ctr)	Abundance (baseMean)	Parent tRNA
TCGTGGGTTCGAGCCCCACGTTGGGCG	27	1.30E-04	6.58	373.86	tRNA-LysCTT
GCATTGGTGGTTCAGTGGTAGAATTCTCGC	30	1.21E-03	3.50	531.33	tRNA-GlyGCC
GCATTGGTGGTTCAGTGGTAGAATTCTCGCCTG	33	3.72E-03	2.47	1376.64	tRNA-GlyGCC
TCGTTTCCCGGCCAACGCA	19	4.04E-03	0.17	19.71	tRNA-GlyTCC
GCATCGGTGGTTCAGTGGTAGAATTCTCGCCT	32	6.31E-03	2.92	15.56	tRNA-GlyGCC
TCCCTGGTGGTCTAGTGGTTAGGATTCG	28	1.52E-02	2.91	162.85	tRNA-GluCTC
AATCTCAAGGTTGCGG	16	2.15E-02	0.27	8.02	tRNA-IleAAT
TCCCTGGTGGTCTAGTGGTTAGGATTCGGCG	31	2.18E-02	2.01	655.25	tRNA-GluCTC
GCGGGTGTAGCTCAGT	16	3.75E-02	0.47	47.75	tRNA-CysGCA
GCGGGTGTAGCTCAGTGGTA	20	4.22E-02	2.23	37.58	tRNA-CysGCA
GGGGGTGTAGCTCAAT	16	4.61E-02	1.95	42.82	tRNA-CysGCA
CGATTCCCGGGCGGCGCA	18	4.86E-02	3.31	14.52	tRNA-GlyCCC

Table S4. Significantly altered host HDL-miRNAs in human atherosclerosis (read level).

miRNA read	Length	P-value	FoldChange (CAC/Ctr)	Abundance (baseMean)	miRNA
ACCCTGTAGATCCGAATTTGT	21	3.88E-03	4.58	78.41	hsa-miR-10a-5p
TGAGATGAAGCACTGTAGCTCA	22	3.96E-03	0.13	16.60	hsa-miR-143-3p
TGTTGTACTTTTTTTTTTGT	20	5.33E-03	6.65	12.89	hsa-miR-3613-5p
AATAATACATGGTTGATCTTT	21	9.13E-03	5.23	19.43	hsa-miR-369-3p
TGTCCCACCCCCACTCCTGTTT	22	2.11E-02	0.26	28.42	hsa-miR-4433b-5p
AAACCGTTACCATTACTGAGT	21	2.82E-02	1.81	612.60	hsa-miR-451a
TGAGGTAGTAGGTTGTGT	18	3.21E-02	1.85	98.80	hsa-let-7b-5p
CTGGCCCTCTCTGCCCTTCCGT	22	3.36E-02	0.27	19.56	hsa-miR-328-3p
AAACCGTTACCATTACTGAGTT	22	3.37E-02	1.91	375.74	hsa-miR-451a
AAACCGTTACCATTACTGAGTTT	23	3.89E-02	2.50	87.89	hsa-miR-451a
TATTGCACTTGTCCCGGCCTG	21	4.57E-02	1.62	228.56	hsa-miR-92a-3p

Table S5. Significantly altered host HDL-rDRs in human atherosclerosis (read level).

rDR read	Length	P-value	FoldChange (CAC/Ctr)	Abundance (baseMean)	Parent rRNA
TCCCTGCCCTTTGTACACACCGCCCGTCGCTACTACCGAT	40	6.19E-06	0.04	32.52	rRNADB_18S_gi
GGAACGTGAGCTGGGTTTAGACCGTCGTGAGA	32	8.97E-05	0.05	31.32	rRNADB_28S_gi
TCCCTGCCCTTTGTACACACCGCCCGTCGCTACT	34	1.00E-03	0.15	29.40	rRNADB_18S_gi
TTCCGGAGAGGGAGCCTGAGAAACG	25	3.49E-03	0.12	25.42	rRNADB_18S_gi
AAAGAAGACCCTGTTGAGCTTG	22	3.64E-03	1.92	167.86	rRNADB_28S_gi
ACCACATCCAAGGAAGGCAGCAGGCGC	27	3.94E-03	7.26	25.14	rRNADB_18S_gi
GGTAAACGGCGGGAGTAACTATGACTCT	28	4.05E-03	4.92	26.01	rRNADB_28S_gi
GGTAGCCAAATGCCTCGTCATCT	23	5.62E-03	0.24	45.20	rRNADB_28S_gi
CCCTGTTGAGCTTGACTCTAGTCT	24	9.17E-03	0.17	19.03	rRNADB_28S_gi
ACTAATAGGGAACGTGAGCTG	21	1.57E-02	2.66	36.10	rRNADB_28S_gi
GGGTTCGATTCCGGAGAGGGAGCCTGAGAAA	31	1.59E-02	7.82	16.59	rRNADB_18S_gi
GATTTCTGCCCAGTGCTCTGAA	22	0.01684	2.03	93.74	rRNADB_28S_gi
CTTTGTACACACCGCCCGTCGC	22	0.01891	0.23	13.70	rRNADB_18S_gi
AAGACCCTGTTGAGCTTGACTCTAGT	26	0.02307	1.98	126.15	rRNADB_28S_gi
TATAGACAGCAGGACGGTGGCCA	23	0.02348	0.28	56.03	rRNADB_28S_gi
GGGAACGTGAGCTGGGTTTAGACCGTCGTGAGACAGG	37	0.02477	0.23	31.68	rRNADB_28S_gi
ATCCTTCGATGTCGGCTCTTCCT	23	0.02517	1.29	576.43	rRNADB_28S_gi
AATCAGCGGGGAAAGAAGAC	20	0.02536	4.68	8.71	rRNADB_28S_gi
GTGATTTCTGCCCAGTGCTCTG	22	0.02941	4.11	15.99	rRNADB_28S_gi
CTTTGTACACACCGCCCGTC	20	0.03058	2.54	16.77	rRNADB_18S_gi
ACTGGGTGCTGTACGCTT	18	0.03179	4.86	10.83	RNA5SP103
GTAGCTGGTTCCCTCCGAAGT	21	0.0325	3.84	17.02	rRNADB_28S_gi
ACGTGAGCTGGGTTTAGACCGTCGTGAGACAGGTTAG	37	0.03341	0.31	55.80	rRNADB_28S_gi
ATTGGAGGGCAAGTCTGGTGCCA	23	0.0354	2.15	68.20	rRNADB_18S_gi
AACGGCGGGAGTAACTATGACTCTCTTAAGGTAGCC	36	0.03595	3.83	31.58	rRNADB_28S_gi
CGTGAGCTGGGTTTAGACCGT	21	0.0369	0.40	31.25	rRNADB_28S_gi
AAGACCCTGTTGAGCTTGACT	21	0.03758	2.01	73.48	rRNADB_28S_gi
AGCGCGGGTAAACGGCGGGAGTAACTAT	28	0.03816	3.71	32.43	rRNADB_28S_gi
TGTGATTTCTGCCCAGTGCTCTGAATG	27	0.03896	2.02	134.47	rRNADB_28S_gi
TTCGATGTCGGCTCTTCCTATCATT	25	0.03899	2.83	37.15	rRNADB_28S_gi
AGGTAGCCAAATGCCTCGTCA	21	0.04152	4.53	12.01	rRNADB_28S_gi
CAATAACAGGTCTGTGATGCCC	22	0.04237	2.74	20.41	rRNADB_18S_gi
CGCGGGTAAACGGCGGGAGTAAC	23	0.04237	0.29	22.42	rRNADB_28S_gi
ACGTGAGCTGGGTTTAGACCG	21	0.04467	0.52	58.09	rRNADB_28S_gi
CTTAAAGGAATTGACGGAAGGGCACCACCAG	31	0.0469	0.33	44.08	rRNADB_18S_gi
GTAAACGGCGGGAGTAACTATGACTCTCT	29	0.04757	2.18	64.72	rRNADB_28S_gi
AACGGCGGGAGTAACTATGACTCTCTTAAGG	31	0.04958	3.56	22.49	rRNADB_28S_gi

Table S7. Sequences of synthesized oligonucleotides used in the study.

Name	Assay	Sequence (5'-3')	Company
tDR-GlyGCC-30; ssRNA-tDR-GlyGCC-30; tDR-GlyGCC-5'-tRH-30; PO-tDR-GlyGCC-30	MST	rGrCrA rUrUrG rGrUrG rGrUrU rCrArG rUrGrG rUrArG rArArU rUrCrU rCrGrC /3AlexF647N/	IDT
tDR-GlyGCC-5'T	MST	rGrCrA TTrG rGTrG rGrUrU rCrArG rUrGrG rUrArG rArArU rUrCrU rCrGrC /3AlexF647N/	IDT
tDR-GlyGCC-5'midT	MST	rGrCrA TTrG rGTrG rGTT rCrArG TrGrG TrArG rArArU rUrCrU rCrGrC /3AlexF647N/	IDT
tDR-GlyGCC-5'mid3'T	MST	rGrCrA TTrG rGTrGrGTT rCrArG TrGrG TrArG rArAT TrCT rCrGrC /3AlexF647N/	IDT
dsRNA-tDR-GlyGCC	MST	Sense:rGrCrArUrUrGrGrUrGrGrUrUrCrArGrUrGrGrUrArGrArArUrUrCrUrCrGrC/3AlexF647N/;<br>Antisense:rGrCrGrArGrArArUrUrCrUrArCrCrArCrUrGrArArCrCrArCrCrArArUrGrC	IDT
2'-O'Me-tDR-GlyGCC	MST	rGrCrA mUmUrG rGmUrG rGmUmUrCrArG mUrGrG mUrArG rArAmU mUrCmU rCrGrC /3AlexF647N/	IDT
tDR-GlyGCC-DNA	MST	GCA TTG GTG GTT CAG TGG TAG AAT TCT CGC /3AlexF647N/	IDT
tDR-GlyGCC-LNA	MST	rGrC+A* rUrU+G* rGrU+G* rGrUrU+C*rA+G* rUrG+G* rUrArG +A*rArU rU+C*rU rCrGrC /3AlexF647N/	IDT
tDR-GlyGC-5'tRF	MST	rGrCrA rUrUrG rGrUrG rGrUrU rCrArG /3AlexF647N/	IDT
tDR-GlyGCC-3'tRF	MST	rUrGrG rUrArG rArArU rUrCrU rCrGrC /3AlexF647N/	IDT
3'tRH-GlyGCC	MST	rCrUrG rCrCrA rCrGrC rGrGrG rArGrG rCrCrC rGrGrG rUrUrC rGrArU rUrCrC /3AlexF647N/	IDT
tDR-GlyCCC-30	MST	rGrCrG rCrCrG rCrUrG rGrUrG rUrArG rUrGrG rUrArU rCrArU rGrCrA rArGrA /3AlexF647N/	IDT
tDR-LysCTT-30	MST	rCrCrG rGrCrU rArGrC rUrCrA rGrUrC rGrGrU rArGrA rGrCrA rUrGrA rGrArC /3AlexF647N/	IDT
tRH-GlyGCC-PS	MST	rG*rC*rA* rU*rU*rG* rG*rU*rG* rG*rU*rU*rC*rA*rG* rU*rG*rG* rU*rA*rG* rA*rA*rU* rU*rC*rU* rC*rG*rC* /3AlexF647N/	IDT
tDR-GluCTC-30	MST	rCrCrU rGrGrU rGrGrU rCrUrA rGrUrG rGrUrU rArGrG rArUrU rCrGrG rCrGrC /3AlexF647N/	IDT
miR-223-3p	MST	rUrGrU rCrArG rUrUrU rGrUrCrArArA rUrArC rCrCrC rA/3AlexF647N/	IDT
miR-375-3p	MST	rUrUrU rGrUrU rCrGrU rUrCrG rGrCrU rCrGrC rGrUrG rA /3AlexF647/	IDT
AS-Scr	MST	ACG TCT ATA CGC CCA	Exiqon
AS-GlyGCC	MST	CAC TGA ACC ACC AAT	Exiqon
tDR-GlyGCC-AF546	EMSA	rGrCrA rUrUrG rGrUrG rGrUrU rCrArG rUrGrG rUrArG rArArU rUrCrU rCrGrC /3AlexF546N/	IDT
tDR-GlyGCC	real-time PCR	TTC TAC CAC TGA ACC ACC AAT GC	Qiagen
ssRNA standard	SYTO	rArGrA rGrArA rCrUrC rGrGrG rUrGrA rArGrG rArArC rU	IDT

Notes: *=LNA (locked-nucleic acid) base; r=RNA nucleotide; m=2-O-methylated nucleotide; 3AlexF647N=3' terminal fluorophore; tRF=tRNA fragment; AS=antisense;
